# Anacardic acid inhibits pancreatic cancer cell growth, and potentiates chemotherapeutic effect by Chmp1A - ATM - p53 signaling pathway

**DOI:** 10.1186/s12906-018-2139-3

**Published:** 2018-02-20

**Authors:** Maiyon Park, Danielle Upton, Melodie Blackmon, Valerie Dixon, Scott Craver, Dawn Neal, Derek Perkins

**Affiliations:** 1 Kentucky College of Osteopathic Medicine, University of Pikeville, Pikeville, KY 41501 USA; 2 Department of Chemistry, University of Pikeville, Pikeville, KY 41501 USA

**Keywords:** Anacardic acid, Ataxia telangiectasia mutated, BXPC-3, Chromatin modifying protein 1A, 5-Fluorouracil, Gemcitabine, p53, PANC-1, Pancreatic cancer

## Abstract

**Background:**

Pancreatic cancer is one of the leading causes of cancer related death and its incidence has risen steadily. Although anticancer drugs have been developed based on the new molecular findings, the drugs have produced unsatisfactory results due to toxicity and resistance. Thus, a complementary therapeutic intervention is urgently needed for pancreatic cancer patients.

**Methods:**

The aim of this study was to assess the potential therapeutic effect of Anacardic acid on pancreatic cancer in vitro and elucidate its underlying mechanisms. Human pancreatic cancer cells were treated with Anacardic acid and assessed for the cytotoxic effect using MTT and spheroid formation assays. Using the same methods, the synergy between Anacardic acid and 5-Fluorouracil or Gemcitabine was determined. To elucidate the underlying molecular mechanisms, Western blot analysis and immunocytochemistry were performed on cancer cells treated with Anacardic acid alone or in combination with 5-Fluorouracil or Gemcitabine. Chromatin Modifying Protein 1A (Chmp1A), Ataxia Telangiectasia Mutated (ATM), and p53 were the primary signaling molecules examined. In addition, Chmp1A was silenced with shRNA to examine the necessity of Chmp1A for the anticancer effect of Anacardic acid, 5-Fluorouracil, or Gemcitabine.

**Results:**

Anacardic acid induced an anticancer effect in pancreatic cancer cell lines in a dose dependent manner, and increased the cytotoxicity of 5-Fluorouracil or Gemcitabine in MTT cell viability assays. In spheroid formation assays, spheroids formed were smaller in size and in number upon Anacardic acid treatment compared to control. Mechanistically, Anacardic acid exerted its anticancer activity via the activation of Chmp1A, ATM, and p53. Interestingly, 5-Fluorouracil and Gemcitabine also induced an increase in Chmp1A protein level, suggesting that Chmp1A might mediate the cytotoxic action of chemotherapeutics. Silencing experiments indicate that Chmp1A is required for the action of Anacardic acid, but not for 5-Fluorouracil or Gemcitabine.

**Conclusions:**

Our data suggests that Anacardic Acid might be a promising complementary supplement to slow the initiation or progression of pancreatic cancer.

## Background

Pancreatic cancer is an extremely aggressive disease that develops from non-invasive precursors, pancreatic intraepithelial neoplasia (PanIN) [1]. Due to great advances in the understanding of pancreatic cancer biology, the molecular mechanisms underlying pancreatic cancer development have been fairly well elucidated. The activating mutation of the Kirsten rat sarcoma 2 (K-Ras), a viral oncogene homolog is one of the earliest event of the PanINs, which increases in frequency as the disease progresses [2]. Epidermal Growth Factor family ligands (TGF-α and EGF) and their receptors (ERBB2, known as HER2/neu, and ERBB3) also function at the earliest stages of pancreatic neoplasia [3]. Mutation of tumor suppressor p53 is a later stage event that is seen in more than 50% of patients with pancreatic adenocarcinoma [4, 5]. As the tumor develops, the SMAD4/DPC4 gene is frequently altered, which is an indicator of poor prognosis in pancreatic adenocarcinoma [6]. Loss of wild type breast cancer gene 2 (BRCA2) also appears in the late-stage of patients who inherited the germ-line heterozygous mutations of BRCA2 [7, 8]. More recently, mutations in Ataxia Telangiectasia Mutated (ATM) have been identified in patients with familial pancreatic cancer [9]. We additionally reported that Chromatin modifying protein 1A (Chmp1A) of the Endosomal sorting complex required for transport (ESCRT) family member plays a role in pancreatic cancer as a tumor suppressor [10–12].

Based on the structure and function, ESCRT family is classified as ESCRT- 0, I, II, and III [13]. They collectively act to transport the membrane-associated proteins such as receptor proteins into lysosomes for degradation via the formation and sorting of multivesicular bodies (MVBs). Chmp1A (called Did2/ Vps 46–1 in yeast) is a member of ESCRT-III and functions in the sorting of MBVs like the other members of ESCRT-III complex [14, 15]. However, Chmp1A is unique since it is the only protein of ESCRT family that contains a nuclear localization signal (NLS) at its N-terminus [12, 16]. In addition to their function in the formation and sorting of MVBs, recent studies have linked ESCRT family with a number of human diseases [17, 18]. Correspondingly, we have provided the first evidence showing that Chmp1A functions as a tumor suppressor in the pancreas via the activation of the tumor suppressor p53 [1]. We next have shown that NLS of Chmp1A is required to facilitate the growth inhibitory function of all-trans retinoic acid (ATRA) [12]. We have further demonstrated that Chmp1A inhibits pancreatic tumor cell growth via the activation of ATM, and that the NLS of Chmp1A is important for the activation of ATM and p53 [11].

New therapeutics have been developed based on these front-line molecular insights and clinically tried on patients. However, the therapeutics have produced disappointing results in clinical trials due to severe toxicity and development of resistance to medication [19]. Thus, there is an urgent need for complementary therapeutic interventions to improve the efficacy of drugs by minimizing drug-mediated toxicity and resistance. To overcome toxicity and resistance of the therapeutics, non-toxic dietary supplement alone or in combination with low doses of therapeutics have been successfully assessed for the treatment of various cancers [20]. Most dietary supplements have derived from plants, which encompass a large number of current medicines [21]. Organic compounds are the biologically active ingredients of plant medicine and these are often isolated from plants or synthesized as analogues for diverse applications. Anacardic acid (AA) is one of the dietary supplements isolated from cashew apple and nut, the fruits of *Anacardium occidentale*, and cashew nutshell liquid (CNSL, products of the tree). AA is also found in mangos [22]. Although collectively referred as AA, cashew products were shown to contain several distinct AAs based on their unique side chains [23].

AA has been shown to exert anti-proliferative activity in cancer cells such as breast, lung, and prostate. AA regulates various signaling pathways for its activities; association with p53 in breast and prostate cancer [20, 24], inhibition of Src/FAK/Rho GTPase to block angiogenesis in prostate cancer [24], and correlation with ATM in squamous cell carcinoma cells of lung [25]. As for chemotherapeutics, 5-FU was shown to activate p53 [26] and ATM in colorectal cancer [27], and GEM to activate p53 in breast cancer [20]. In addition, we have shown that Chmp1A activates ATM and p53 in pancreatic cancer cells [10–12]. Based on these mechanistical findings, we have proposed that AA exhibits anticancer activity via the activation of Chmp1A, ATM and p53, which we refer to as the Chmp1A - ATM - p53 signaling pathway. We also propose that AA increases the anticancer efficacy of 5-FU and GEM, possibly by activating the same signaling pathway. In summary, the results of this study will provide the functions and the underlying mechanisms of new complementary medicine, which could be applied to the prevention and treatment of pancreatic cancers.

## Methods

### Reagents

Dimethyl sulfoxide (DMSO) was obtained from Thermo Fisher Scientific, MA, USA, Puromycin dihydrochloride, 5-Fluorouracil, and Gemcitabine hydrochloride from R & D system, Minneapolis, MN, USA, and Anacardic acid from EMD Millipore, Germany. Polyclonal antibody against Chmp1A was generated in our laboratory and successfully used [10–12]. Other antibodies were purchased from commercial sources: rabbit polyclonal antibodies against p53, and monoclonal antibody against phospho- p53 at serine 15 from Cell Signaling, USA; monoclonal antibodies against Gapdh and phospho- ATM at serine 1981 from Pierce, USA; monoclonal antibody against ATM from Sigma-Aldrich, Germany. Goat anti-rabbit or mouse HRP conjugated secondary antibody were purchased from Chemicon-EMD Millipore, Germany.

### Cell culture

All cell lines were obtained from American Type Culture Collection (ATCC, Manassas, VA, USA). BXPC-3 cells were cultured in RPMI medium containing 10% fetal bovine serum (FBS), and 10,000 U/mL of penicillin-streptomycin. CAPAN-2, BXPC-3, and PANC-1 cells were cultured respectively in McCoy 5A, RPMI-1640, and Dulbecco’s modified Eagle’s medium (DMEM) that contain 10% FBS, and 10,000 U/mL of penicillin-streptomycin. Chmp1A silenced PANC-1 cells were maintained in DMEM media supplied with 10% FBS, 10,000 U/mL of penicillin-streptomycin and 1 μg/ml puromycin as described previously [10]. All the cells were cultured at 37 °C under 5% CO2. The antibiotics, FBS and media were obtained from Invitogen- Thermo Fisher Scientific, USA. Fluorescence coupled secondary antibodies were obtained from Molecular Probes- Thermo Fisher Scientific, USA; Alexa Flour 488 and Alexa Flour 555 for green and red fluorescence, respectively.

### MTT assays

AA, GEM and 5-FU were dissolved in DMSO as 100 μM stock and kept in the dark as recommended by the manufacturer. 30,000–70,000 cells per well were seeded onto wells in 24- well plate. Twenty four hours later the media were replaced with fresh media containing AA, 5-FU or GEM alone or in combination of AA and 5-FU or GEM, and cells were incubated at 37 °C. The doses were adjusted based on the published data [20, 25]. MTT reagent (Sigma-Aldrich, Germany) was dissolved in PBS, filtered, and kept at 4 °C in dark. MTT assays were performed following the manufacturer’s instruction with a few modifications. Briefly, 24, 48, and 72 h later, 50 μl of MTT was added to each well and incubated for 4 h at 37 °C. The MTT precipitate was dissolved by pipetting up and down after addition of 500 μl of color development reagent (isopropanol plus 0.04 N HCl), and the optical density (OD) was measured at 590 nm (620 nm as reference) with a BioTek EON™ Spectrophotometer (Thermo Fisher Scientific, USA).

### 3 dimensional spheroids formation assays

To mimic a 3- dimensional (3D) in vivo tumor environment, we cultured cells onto the Nunclon™ Sphera plates (Thermo Fisher Scientific, USA) that promote spheroid formations. Following manufacturer’s instructions, same number of cells was seeded onto the plates. The next day, the cells were treated with the appropriate reagent(s). Every other day, half of the media was replaced with fresh media containing appropriate reagent(s), and photos were taken 7 days after initial treatment using an Olympus 1X71 inverted microscope. The number of spheroids was counted, and the diameter of spheroid was measured. DMSO treated samples were used as a control.

### Western blot analysis

We processed cells between 1 and 2 days after treatment with AA, 5-FU or GEM for WB analysis. WB was carried out following the protocol established in the lab by lysing cells with SoluLyse-M protein extraction reagent (Genlantis, CA, USA) containing protease inhibitor cocktail (Sigma-Aldrich, Germany) [10–12]. BCA assay (Pierce- Thermo Fisher Scientific, USA) was performed to measure protein concentration on Microplate Spectrophotometer (BioTek EON™, UK). TGX™ precast gels (BioRad, CA, USA) were used to separate proteins, which were transferred to nitrocellulose membranes (Pierce-Thermo Fisher Scientific, USA). After incubation with primary and secondary antibody, the membranes were processed with chemiluminescence substrate (Pierce-Thermo Fisher Scientific, USA) and protein bands were visualized using ChemiDoc™ XRS imaging system (Bio-Rad, CA, USA).

### Immunocytochemical analysis

For immunocytochemical analysis, we seeded cells over coverslips onto 12- wells plate, and treated cells with appropriate reagent(s) the next day. Within 2 days after treatment, the cells were fixed with 4% para-formaldehyde for 15 min, permeabilized with series of 75%, 50% and 25% of MeOH in PBS for 5 min each, and rehydrated with PBS, 3 times for 10 min each. The cells were incubated with blocking buffer (PBS/5% serum/0.1% Tween 20) for 30 min, and with primary antibody overnight at 4 °C. Next day, the cells were washed with PBS and incubated with fluorescence labeled secondary antibody till the protein expression was apparent. The cell- bound coverslips were mounted on the slides using Vectashield mounting media with Dapi nuclei marker (Vector Laboratory, INC, USA), and analyzed with Olympus FluoView FV1000 confocal microscope.

### Silencing of Chmp1A protein

We generated stable cell lines with Chmp1A- specific shRNA (KD6) to silence Chmp1A protein. KD6 is one of the clones generated and have been used to knockdown (KD) Chmp1A protein. These stable cells were cultured in media containing puromycin following a previously described protocol [10]. In these assays, we increased the amount of AA to induce substantial growth inhibition. And we tested whether Chmp1A is required for the growth inhibitory effect of AA. If Chmp1A is essential for the growth inhibitory function of AA, we hypothesize that AA would not induce growth inhibition in the absence of Chmp1A protein. Clone of cells expressing non-specific shRNA (NS2) was used as a control.

### Statistical analysis

All the assays were performed in triplicates at three biologically independent times. Results from quantitative studies were expressed as Mean ± SD (standard deviation). Most of the comparisons between experimental and control groups were performed by one-way analysis of variance (ANOVA) followed by one-sided Student’s t-test. A *p*-value less than 0.05 was considered statistically significant.

## Results

### AA induces a significant growth inhibition in pancreatic cancer cells

**Fig. 1:**
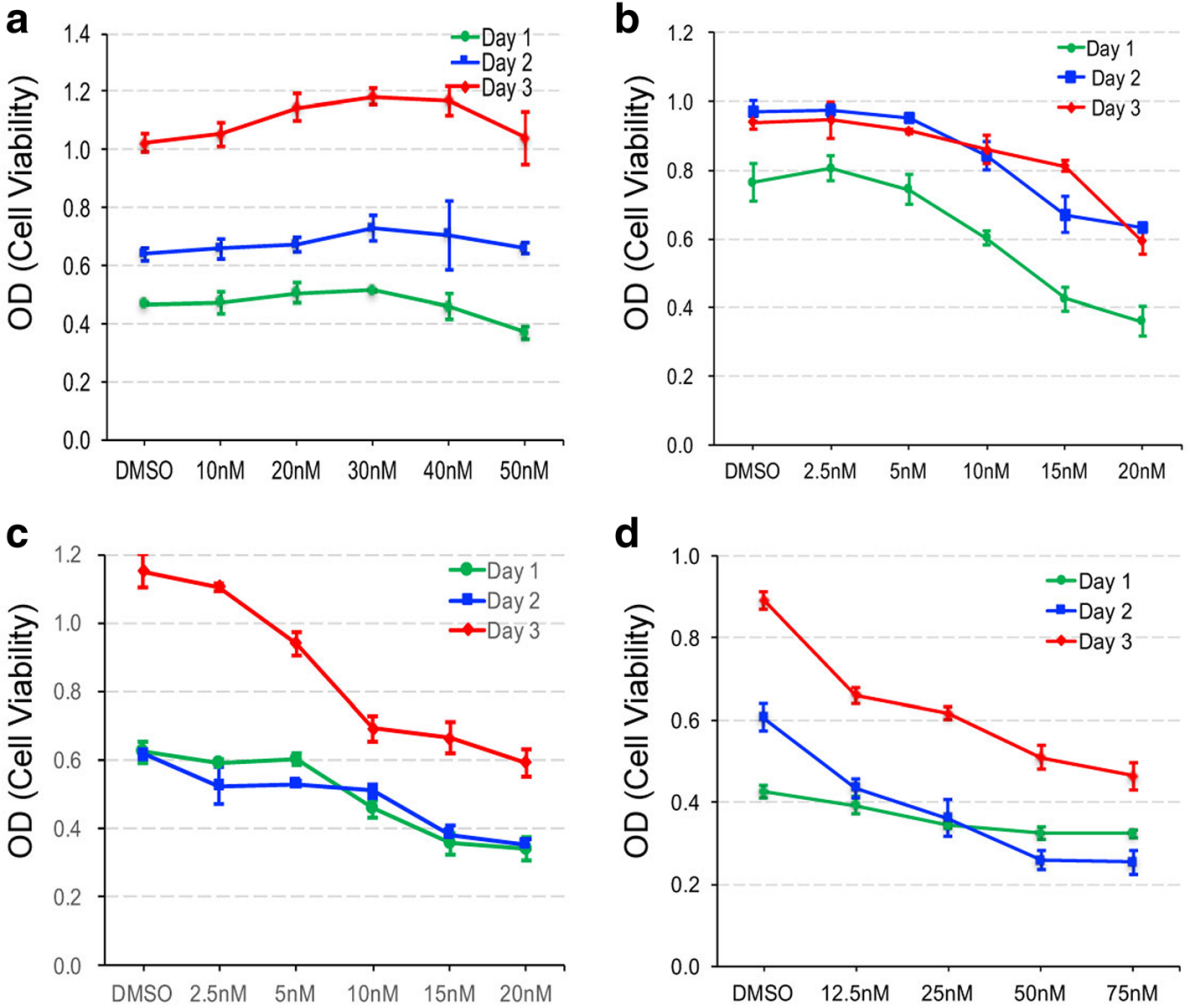
AA induces a dose - dependent growth inhibition in pancreatic cancer cells. Panel **a** shows data obtained from HEK 293, (**b**) from CAPAN-2, (**c**) from BXPC-3, and (**d**) from PANC-1 cells. Label on the X-axis indicates treatment with various concentration of AA in nM or DMSO as control. Data was analyzed from three samples at 24, 48 and 72 h after initial treatment. Error bars indicate standard deviation (SD). *P* value between DMSO control and various doses of AA was < 0.05. P value between each day was also < 0.05 (**b**, **c**, **d**)

To examine the extent of the cytotoxic effect of AA on pancreatic cancer cells, cell viability assays were performed using MTT in three pancreatic cancer cell lines, and HEK 293 cells as control for AA and DMSO treatment. CAPAN-2, BXPC-3, and PANC-1 cells are derived from well differentiated, well to moderately differentiated, and poorly differentiated adenocarcinomas of human pancreas, respectively [28]. Forty thousand to fifty thousand cells were seeded onto 24 well plates for the data shown. Twenty four well, not 96 well plates were used to reduce any error derived from cell number differences between samples. Although minor inhibition was detected on day 1 at 50 nM AA treatment, control HEK 293 cells did not show substantial growth inhibition with AA or DMSO treatment for a period of 3 days (Fig. 1a). However, pancreatic cancer cells showed a significant growth inhibition in a dose dependent manner for a period of 3 days. In CAPAN-2 cells, when examined up to 20 nM, AA showed a major cytotoxicity on day 1, which was tapered slightly from day 2 (Fig. 1b). Similar to CAPAN-2, BXPC-3 cells responded well to AA at the same doses for a period of 3 days (Fig. 1c). Unlike CAPAN-2 cells, the greatest growth inhibition was detected 3 days after the initial treatment in BXPC-3 cells (Fig. 1c). PANC-1 cells, derived from the most malignant cancer of the three, required higher amount of AA to produce substantial growth inhibition (1D), compared to CAPAN-2 or BXPC-3 cells (1B, C). We also examined the effect of AA on a varying number of cells, and found out that the effect of AA is partially dependent on the number of cells plated. Higher amount of AA was required to produce similar cytotoxic effect on samples of more cells compared to samples of less (data not shown). We observed a complete cell death when about 40,000 pancreatic cancer cells were plated onto each well in 24 well plates and treated with 100 nM of AA (data not shown).

### AA potentiates the anticancer effect of 5 - fluorouracil (5-FU) and gemcitabine (GEM)

**Fig. 2:**
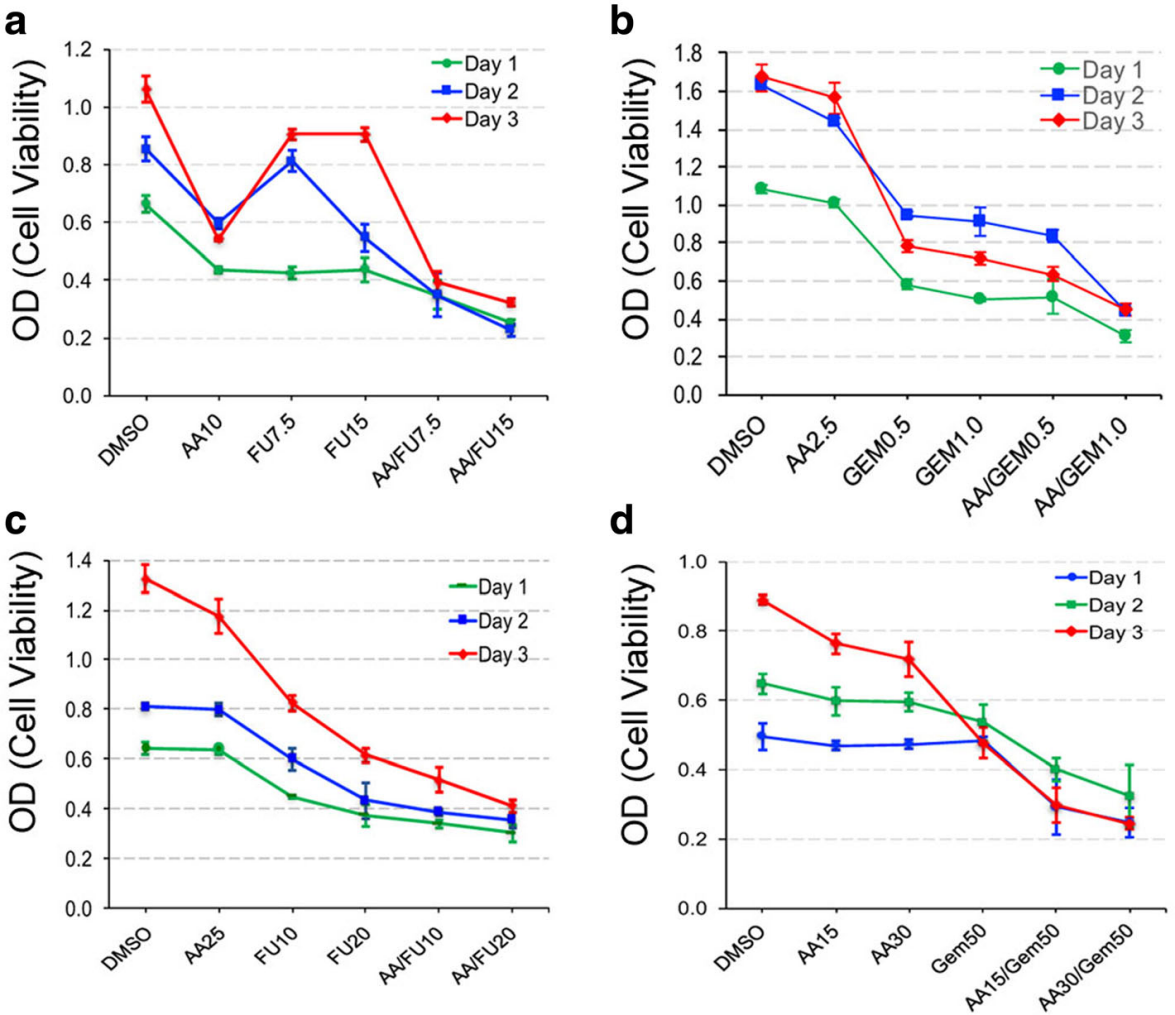
AA increased the cytotoxic effect of 5-FU and GEM. The MTT cell viability data is obtained from BXPC-3 (**a** and **b**) and PANC-1 (**c** and **d**) cells, respectively. Label on the X-axis indicates treatment with various concentration of AA, 5-FU (FU) or GEM alone or in combination of AA and 5-FU or GEM in nM or DMSO as control. Data was analyzed from three samples at 24, 48 and 72 h after initial treatment. Error bars represent standard deviation (SD)

**Fig. 3:**
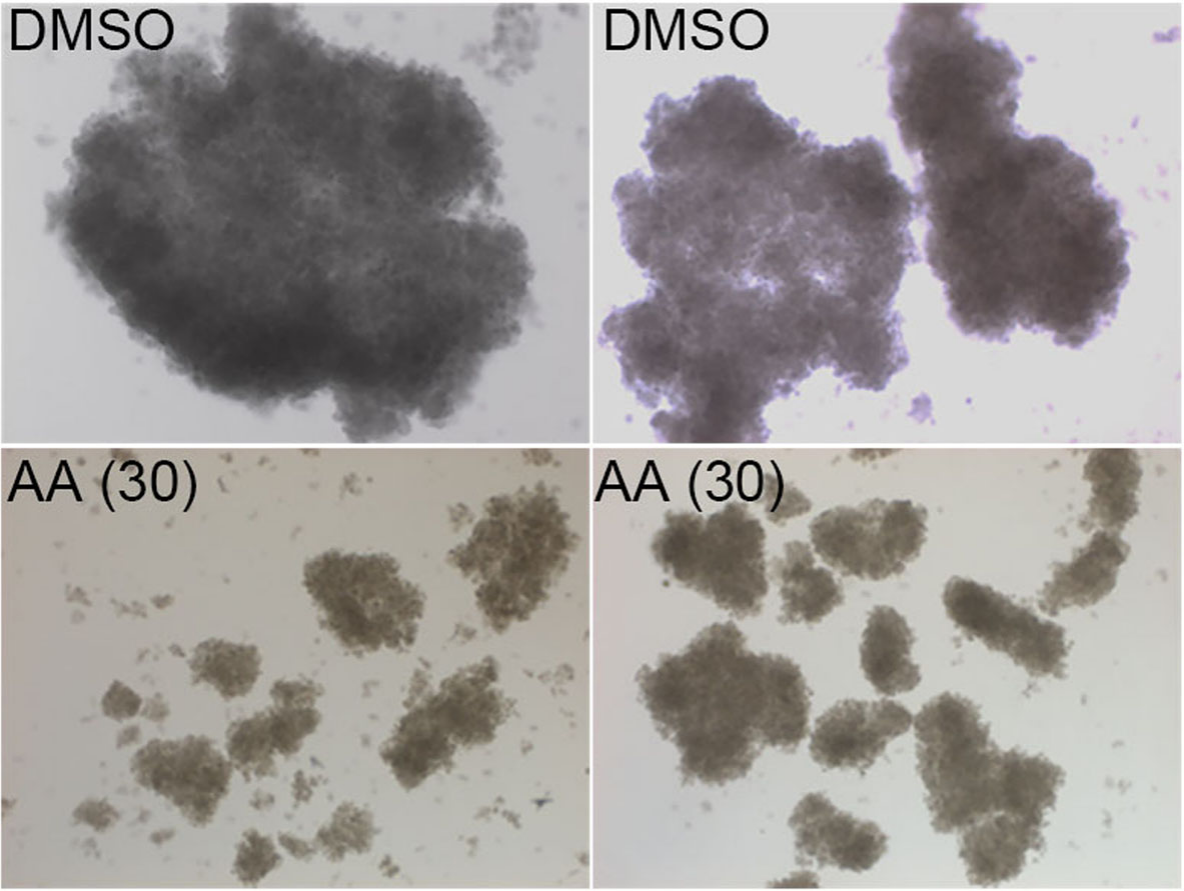
AA induced a decrease in the number and size of spheroids in PANC-1 cells. The same number of cells were seeded onto Nunclon™ Sphera plates. Next day, media was replaced with fresh media containing either 3 μl of DMSO or 3 μl of AA stock solution in 10 ml media to make the final concentration of AA at 30 nM. Every other day, half of the media was replaced with fresh media containing respective reagent. 7 days after the initial treatment, photos were taken from two independent assays

5-FU and GEM are the current chemotherapeutics used for the treatment of advanced pancreatic cancer, and clinical trials have demonstrated that they show similar advantages for survival [29]. We examined whether AA increased the anticancer activity of 5-FU or GEM by treating cells with 5-FU or GEM individually or with AA plus 5-FU or GEM. As shown in Fig. 2a, AA and 5-FU exhibited similar growth inhibition in BXPC-3 cells on day 1, which was additionally inhibited with the combination of the two (see blue line). On day 2 and 3, AA still displayed significant growth inhibition, but 5-FU did not, especially on day 3 (see red line). However, cells treated with a combination of AA and 5-FU showed significantly greater growth inhibition on days 2 and 3, compared to individual AA or 5-FU treatment. GEM was more effective than 5-FU, and induced significant growth inhibition at very low doses, which was further decreased with the addition of AA (Fig. 2b). As for PANC-1 cells, AA and 5-FU each exhibited moderate growth inhibition at the doses examined. The cell growth was further inhibited with the combination of AA and 5-FU (Fig. 2c). AA or GEM treatment induced minor growth inhibition up to day 2, but moderate inhibition on day 3 (Fig. 2d). With combined treatment of AA and GEM, however, the cell growth was significantly inhibited compared to AA or GEM individual treatment (Fig. 3d).

### AA produces growth inhibition and potentiates the anticancer activity of 5-FU or GEM in 3- dimensional spheroid formation assays

Three dimensional (3D) spheroid formation assay has become a standard system for determining the efficacy of anticancer agents since it better represents the in vivo tumor environment compared to two dimensional cell culture [30]. Thus, the anticancer effect of AA was assessed in 3D cell culture, alone or in combination with 5-FU or GEM. When PANC-1 cells were treated with 30 nM of AA, smaller colonies were formed compared to DMSO control (Fig. 3a). At lower than 30 nM of AA, there was little difference in the size of spheroids formed between AA and control treated samples. However, these PANC-1 cells produced fewer number of spheroids: approximately 22% fewer colonies compared to control (Table 1). Compared to control or AA treated cells, 5-FU or GEM treated cells produced significantly smaller spheroids in three biological independent assays: colony diameters of 0.1–0.2 mm in 5-FU or GEM, compared to 0.8–1.2 mm in controls or AA treated cells. When treated with AA plus 5-FU or GEM, similar sizes of spheroids were formed but with smaller number; 50% fewer colonies with AA plus 5-FU, compared to 5-FU alone, and 29% fewer colonies with AA plus GEM compared to GEM alone (Table 1).

**Table 1:** AA increased the effect of 5-FU and GEM in 3D spheroid formation assays

Treatment	Number of Colonies	Size of Colonies (mm)
DMSO	41	0.8–1.2
AA (20 nM)	32	0.8–1.2
5-FU (5 nM)	10	0.1–0.2
5-FU/AA	5	0.1–0.2
GEM (5 nM)	7	0.1–0.2
GEM/AA	5	0.1–0.2

The same number of cells were seeded onto Nunclon™ Sphera plates. Next day, media was replaced with new media containing the following reagents as described in the table. Every other day, media was replaced with fresh media containing respective reagent(s). 7 days after the initial treatment, the number of spheroids was counted, and the size of spheroids measured in diameter. The data was collected from two independent assays

### AA, 5-FU, and GEM induce an activation of Chmp1A - ATM - p53 signaling pathway

**Fig. 4:**
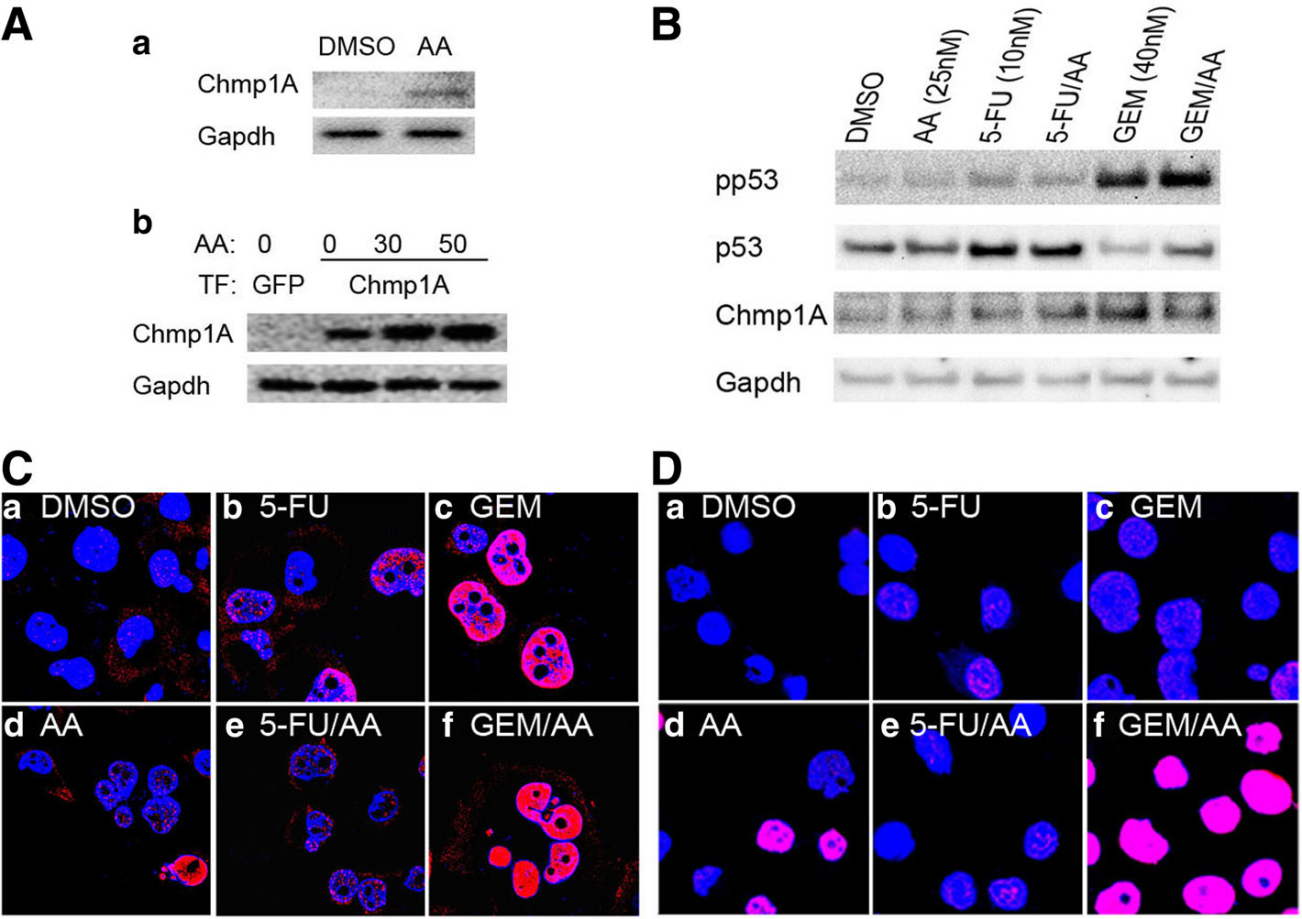
AA induces an increase in Chmp1A protein level and an activation of ATM and p53 in PANC-1 cells. Western blot analysis showed an increase in Chmp1A protein upon AA (30 nM) treatment in control cells (**A***a*), and in Chmp1A transfected cells (**A***b*). GFP was transfected as control for Chmp1A transfection (**A***b*). 5-FU and GEM alone or in combination with AA produced an increase in Chmp1A protein, and activation of p53 (**B**). Gapdh was used as loading control. Immunocytochemistry demonstrated the activation of ATM (pATM) and p53 (pp53) in (**C** and **D**), respectively. AA, 5-FU or GEM alone or AA in combination with 5-FU or GEM treatment caused an activation of ATM and p53. Notice that all the cells treated with AA plus GEM had strong activation of ATM (**C***f*) and p53 (**D***f*), compared to individual treatment with GEM (**C***c* for ATM and **D***c* for p53) or AA (**C***d* for ATM and **D***d* for p53). Cells are processed equally for immunostaining, and the images were captured using the same settings. In (**C** and **D**), blue stands for Dapi positive nuclear expression, red for phospho-ATM or phospho-p53, and pink for co - expression of blue and red

Our previous studies have shown that Chmp1A functions as a pancreatic tumor suppressor via the activation of ATM and p53 [10–12]. Because AA, 5-FU and GEM are known to alter the signaling activity of ATM and/or p53 [20, 24–27], we examined whether AA, 5-FU or GEM activated Chmp1A - ATM - p53 signaling pathway by examining the protein levels of Chmp1A, and the phosphorylated form of ATM and p53. As shown in Fig 4Aa, AA treated PANC-1 cells showed an increase in Chmp1A protein level compared to control. We additionally examined the effect of AA on Chmp1A protein level in Chmp1A overexpressing PANC-1 cells. Compared to control GFP expressing cells, cells overexpressing Chmp1A showed an increase in Chmp1A protein (Fig. 4Ab). When the Chmp1A overexpressing cells were treated with 30 nM or 50 nM of AA, Chmp1A protein expression was profoundly increased compared to control DMSO treated cells (Fig. 4Ab). Next, cells were treated with AA, 5-FU or GEM individually or AA in combination with 5-FU or GEM, and the protein level of Chmp1A, p53, and phospho- p53 was examined. Chmp1A protein levels were slightly increased by AA and 5-FU individual treatment, but this increase was not enhanced by the combination of the two. Compared to 5-FU or 5-FU plus AA, GEM and GEM plus AA had greater effect on the Chmp1A protein expression (Fig. 4B). An increased level of p53 protein was detected in the cells treated with 5-FU or 5-FU plus AA, but not with GEM or GEM plus AA. Phospho- p53 (active form) was increased slightly with AA, and more with 5-FU or 5-FU plus AA, but significantly more with GEM or GEM plus AA treatment (Fig. 4B). BXPC-3 cells showed a similar pattern in an increase in Chmp1A, and activation of ATM and p53, an activation of Chmp1A - ATM - p53 signaling pathway (data not shown). All the data from Western blot to determine signaling activity were obtained about 36 h after the initial treatment when significant increase in protein level was detected.

Immunocytochemistry was used to examine the activation of ATM and p53 by detecting cellular expression of phospho- specific ATM and p53. AA induced an evident activation of ATM in a few cells compared to control DMSO, supported by phosphorylated- ATM expression (Fig. 4C). ATM activation was visualized in the fraction of cells treated with 5-FU, but was not intensified by the addition of AA (Fig. 4C). GEM treatment induced a robust activation of ATM in most cells treated, which was further enhanced in all the cells treated with GEM plus AA (Fig. 4C). As for the activation of p53, PANC-1 cells treated with AA showed an increased expression of phospho- p53 in the fraction of cells examined (Fig. 4D). Consistent with Western blot analysis, 5-FU alone induced a slight activation of p53, which was not enhanced by the combination of 5-FU and AA (Fig. 4D). Cells treated with GEM showed a modest activation of p53 in most of the cells examined. However, the p53 activation became much more robust with the combined treatment of GEM and AA (Fig. 4D). We did not see any major change in non-phosphorylated form of ATM or p53 expression in PANC-1 cells, and BXPC-3 cells showed similar pattern in the activation of Chmp1A - ATM - p53 signaling pathway (data not shown).

### Chmp1A is required for the anticancer action of AA

**Fig. 5:**
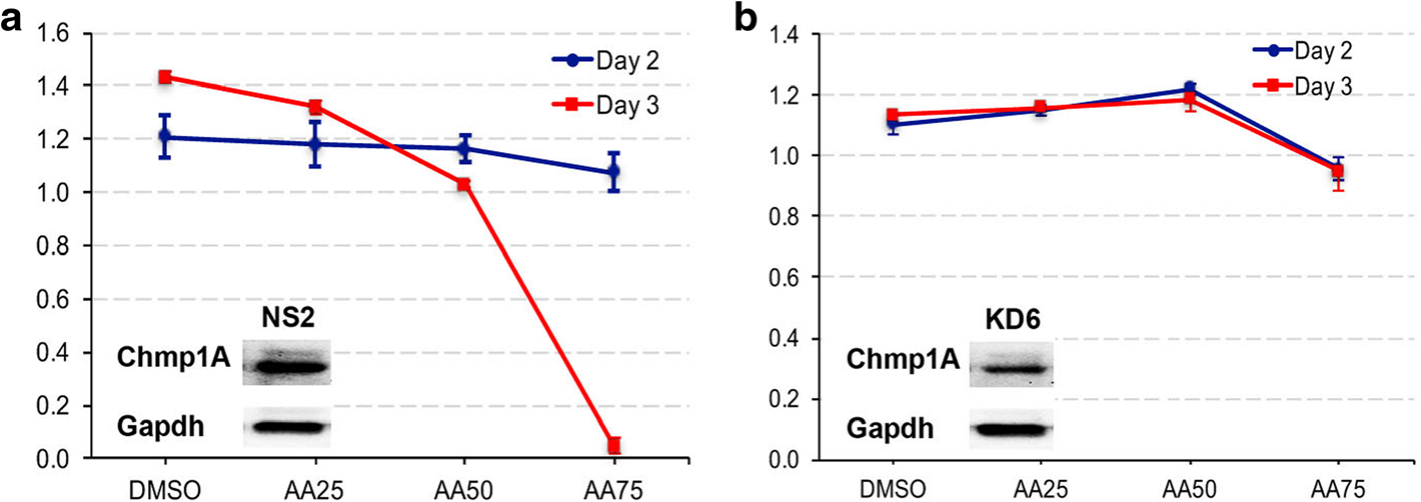
Chmp1A is required for the cytotoxic effect of AA. Panels show control shRNA (NS2, (**a**) and Chmp1A specific shRNA (KD6, (**b**) expressing PANC-1 cells. Control cells showed significant growth inhibition with AA treatment on day 3, compared to control DMSO (**a**). However, Chmp1A silenced cells showed slight growth promotion with AA treatment at 25 and 50 nM, but growth inhibition at 75 nM on both days (**b**), minor though compare to that at 75 nM in control on day 3 especially (red line in **a**). Insets showed the validation of decreased Chmp1A protein in Chmp1A silenced KD6 cells (**b**), compared to control silenced NS2 cells (**a**). Blots for NS2 and KD6 are from the same Western blot. MTT data was acquired from three samples at 48 and 72 h after initial treatment. Error bars represent standard deviation (SD)

Since our data demonstrates that AA exerts its anti-proliferative function by increasing Chmp1A protein level, we examined whether Chmp1A was required for the growth inhibitory function of AA. We hypothesize that AA would not induce growth inhibition in the absence of Chmp1A protein if Chmp1A is required for the growth inhibitory function of AA. To test our hypothesis, we silenced Chmp1A protein level by using clone of cells that stably express Chmp1A specific shRNA (KD6), or non- specific shRNA (NS2) as control. These clones were successfully used in previous reports [10–12]. In this assay, higher amount of AA was used to induce maximum growth inhibition and the effect of Chmp1A silencing on growth inhibition was tested. The reason of using higher amount of AA was that at low concentration, AA might not induce significant growth inhibition to clearly show the dampening effect of Chmp1A silencing on growth. As shown in Fig. 5a, control shRNA expressing PANC-1 cells (NS2) exhibited considerable cell growth inhibition on day 3 upon treatment of AA at 50 or 75 nM. In contrast, Chmp1A specific shRNA expressing PANC-1 cells (KD6) showed a slight growth promotion rather than inhibition with AA treatment at 25 or 50 nM (Fig. 5b). At 75 nM of AA though, Chmp1A silenced PANC-1 cell growth was inhibited compared to DMSO treated controls. However, the growth inhibition shown in Chmp1A shRNA expressing cells at 75 nM was much less substantial compared to that shown in control shRNA expressing cells on day 3 (compare red line in Fig. 5b to a). We performed MTT assays on day 2 and 3, but not on day 1, since we learned that it took time for the Chmp1A shRNA to have effect on the growth from previous studies [10–12].

Since 5-FU and GEM treatment induced an increase in Chmp1A protein level, we tested whether Chmp1A is required for the anticancer effect of 5-FU or GEM using the same shRNA technology described above. Upon 5-FU or GEM treatment, Chmp1A shRNA expressing cells did not exhibit major difference in growth inhibition compared to controls, indicating that Chmp1A is not required for the anticancer action of these therapeutics (data not shown).

## Discussion

In this study, we examined the potential application of Anacardic acid as complementary medicine for pancreatic cancer by treating cells with AA alone or in combination with chemotherapeutics. AA has been shown to be a potential complementary medicine for breast and prostate cancers [31], and this study demonstrates the similar paradigm in pancreatic cancer. Our data reveals that AA inhibits pancreatic tumor growth in 2D cell growth and 3D spheroid formation assays. The effect of AA on growth inhibition of pancreatic cancer was substantial especially in 2D cell cultures. However, the effect of AA on growth was somewhat dependent on the stage of tumor progression and on the number of cells treated. To achieve the similar growth inhibition, a higher amount of AA was required for the cells derived from more progressed compared to less progressed tumors. Also, for similar growth inhibition, higher amount of AA was required for the samples with more cells compared to less cells. This might imply that AA would benefit patients the most if it is incorporated in daily diet as prevention or therapeutic regimen for early stage of pancreatic cancer.

Dietary supplement in combination with chemotherapeutics has been assessed for the enhanced effect on various cancers in vitro, pre-clinical and clinical studies. Those studies have shown promising results for slowing down tumor growth and/or increasing survival rates [32–34]. Thus, we investigated whether AA potentiates the anticancer activity of 5-FU or GEM, current chemotherapeutics for pancreatic cancer. Pancreatic cancer cells exhibited significant growth inhibition when 5-FU or GEM was applied individually in 2D cell growth and 3D spheroid formation assays. The chemotherapeutic induced growth inhibition was further increased when AA was added simultaneously. In summary, this study has provided strong evidence supporting that AA might be used as complementary or alternative medicine for the prevention or treatment of pancreatic cancer.

We then investigated whether Chmp1A is mechanistically involved in the growth inhibitory action of AA, 5-FU or GEM in pancreatic cancer cells. We have shown that Chmp1A suppresses pancreatic cancer cell growth by the activation of ATM and p53 [10–12]. The anticancer activity of AA is shown to be associated with ATM and/or p53 [20, 24–27]. Thus, we assessed Chmp1A, ATM or p53 proteins for their protein level and/or activation in the cells treated with AA, 5-FU, and GEM. AA treated cells have shown an increase in Chmp1A protein level, which was greatly intensified with Chmp1A overexpression, demonstrating the implication of Chmp1A in AA mediated growth inhibition. Unexpectedly, 5-FU and GEM treated cells also have exhibited an increase in Chmp1A protein level, a slightly greater increase than that in AA treated cells. This is the first data to show a molecular link between Chmp1A and the anti- cancer action of 5-FU or GEM. Further, our data on Chmp1A silencing experiments demonstrate that Chmp1A is necessary target signaling molecule for the growth inhibitory action of AA. However, silencing data did not show the necessity of Chmp1A for the anticancer activity of 5-FU or GEM, indicating the difference in signaling pathway between AA and chemotherapeutics.

We also have examined the activation of ATM and p53 using phosphorylated- ATM or p53 antibodies in immunocytochemical assays. Minor activation of ATM was noticed in the subset of cells treated with AA or 5-FU, but major activation in all the cells treated with GEM. With the addition of AA, 5-FU did not show a major change in ATM activation, but GEM showed a greater activation of ATM in all the cells examined. Our data on p53 activation in immunostaining was similar to that obtained from Western blot analysis.

## Conclusions

**Fig. 6:**
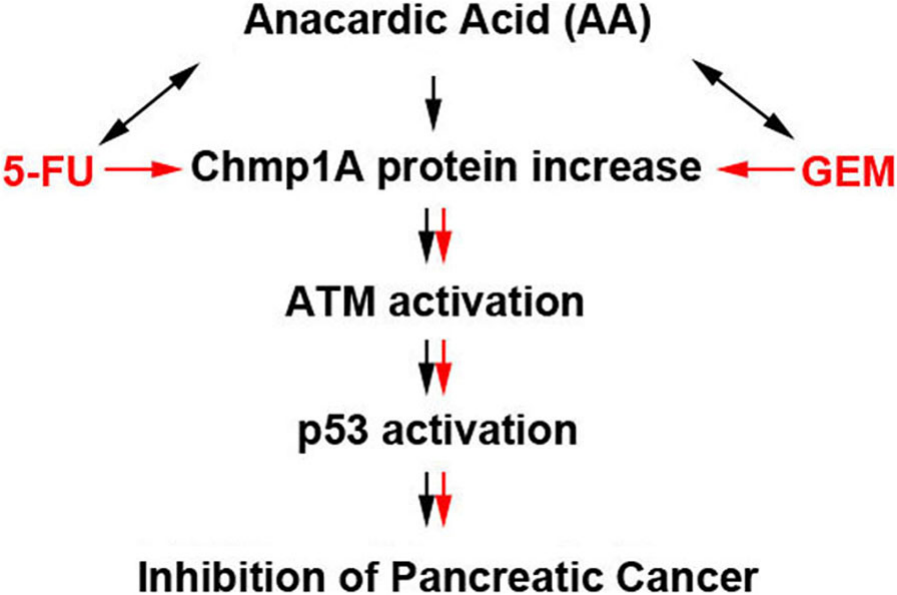
Schematic diagram showing the working hypothesis of Anacardic Acid. As shown in the diagram, AA inhibits pancreatic cancer growth by the increase of Chmp1A protein, which leads to phosphorylation of ATM, an indication of activation. Phospho- ATM then induces activation of p53 via phosphorylation of p53 (shown in short black arrows). 5- FU or GEM may utilize the similar signaling pathway as AA for its anticancer action, increasing Chmp1A protein to activation of ATM and p53 (shown in short red arrows). Subsequently, AA synergizes with 5-FU or GEM (indicated as double headed black arrows), and increases the activation of signaling pathway and cancer cell grwoth inhibition (shown as black and red arrows together). Black arrows indicate signaling activities for both sufficient and necessary action, and red for sufficient action only

Our data provides supporting evidence that AA inhibits pancreatic cancer cell growth via the increase in Chmp1A followed by the activation of ATM and p53, which we refer as Chmp1A - ATM - p53 signaling pathway in this paper (see Fig. 6). The data also provides supporting evidence that the signaling activation becomes augmented with the combination of AA and 5-FU or GEM. This is the first study showing that Chmp1A is a sufficient and necessary target molecule for the anticancer action of AA. It is also the first report demonstrating the sufficient, but not required function of Chmp1A in the mediation of anticancer action of 5-FU and GEM in pancreatic cancer. The results of this study could have significant implications for pancreatic cancer patients, as the addition of Anacardic acid to the current treatment regimen may provide a safe and effective way to improve treatment efficacy. Since our data is obtained from in vitro studies using pancreatic cancer cells, preclinical and clinical studies are required to evaluate the efficacy of complementary interventions with AA for pancreatic cancer.

## Abbreviation

3D: 3- Dimensional
5-FU: 5-Fluorouracil
AA: Anacardic acid
ATM: Ataxia telangiectasia mutated
Chmp1A: Chromatin modifying protein 1A
DMSO: Dimethyl sulfoxide
ESCRT: Endosomal sorting complex required for transport
FBS: Fetal bovine serum
GEM: Gemcitabine
KD6: Chmp1A specific shRNA expressing clone 6
MTT: 3-[4,5-dimethylthiazol-2-yl]-2,5 diphenyl-tetrazolium bromide
nm: Nanometer
nM: Nanomole
NS2: Non-specific, scrambled shRNA expressing clone 2
OD: Optical density
pATM: Phospho- ATM
pp53: Phospho- p53
shRNA: Short hairpin RNA
WB: Western blot
μM: Micromole
